# Supplementary material for: Defect in hematopoiesis and embryonic lethality at midgestation of Vps13a/Vps13c double knockout mice
Source: bioRxiv. 2025 May 13:2025.05.09.653147. Preprint. [Version 1] doi: 10.1101/2025.05.09.653147 (PMC12132566; doi:10.1101/2025.05.09.653147)

bioRxiv preprint doi: https://doi.org/10.1101/2025.05.09.653147; this version posted May 13, 2025. The copyright holder for this preprint (which was not certified by peer review) is the author/funder, who has granted bioRxiv a license to display the preprint in perpetuity. It is made available under aCC-BY 4.0 International license.

Figure S1. Generation *Vps13a*/*Vps13c* double knockout mice. (A) Schematic overview of the process used for the generation of *Vps13a* KO mice to be mated to previously generated *Vps13c* KO mice. *Vps13a*$^{\text{tm1a}}$ mice were first mated to Flp recombinase expressing mice to remove the LacZ and Neo cassettes to generate *Vps13a* conditional KO mice. Subsequently, mice were bred with β-actin-Cre expressing transgenic mice to remove exon 6 and generate constitutive full body *Vps13a* KO mice. (B) Anti-VPS13A western blots of lysates of cortical tissues from both WT and *Vps13a* KO mice confirming absence of VPS13A in the KO mice. (C) Genotyping results of adult mice and E12.5 embryos derived from *Vps13a*$^{+/-}$/*Vps13c*$^{-/-}$ intercrosses. Values in the brackets indicate the number of expected mice/embryos based on Mendelian distribution.

Figure S2. (A) Viability of cells derived from fetal livers assessed by trypan blue staining. Each dot represents one fetal liver with the specific genotype. (B) Percentage of each erythroid subset within the lineage negative gate. Each dot represents one fetal liver with the specific genotype. Results are presented as average plus SD of each subtype of erythroid cells. (C) Bar plot showing

bioRxiv preprint doi: https://doi.org/10.1101/2025.05.09.653147; this version posted May 13, 2025. The copyright holder for this preprint (which was not certified by peer review) is the author/funder, who has granted bioRxiv a license to display the preprint in perpetuity. It is made available under aCC-BY 4.0 International license.

top 15 significantly upregulated (KEGG; top panel) and significantly downregulated (MSigDB; bottom panel) pathways based on adjP comparing DKO S0 cells versus WT.

bioRxiv preprint doi: https://doi.org/10.1101/2025.05.09.653147; this version posted May 13, 2025. The copyright holder for this preprint (which was not certified by peer review) is the author/funder, who has granted bioRxiv a license to display the preprint in perpetuity. It is made available under aCC-BY 4.0 International license.

Fig. S1

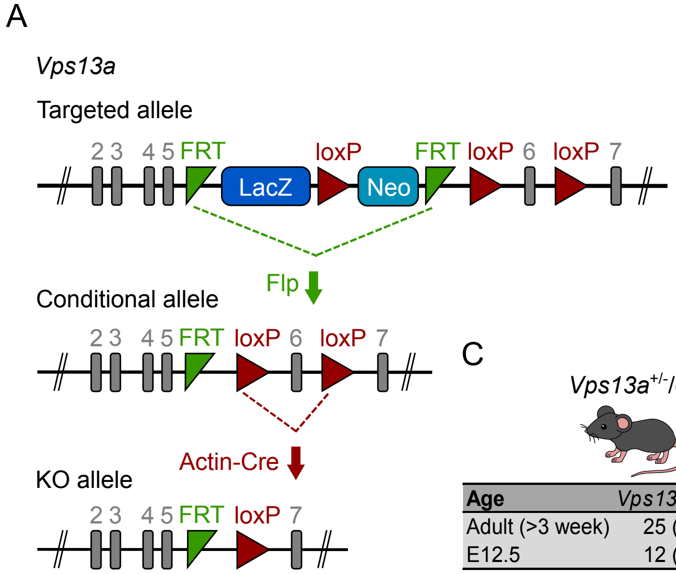

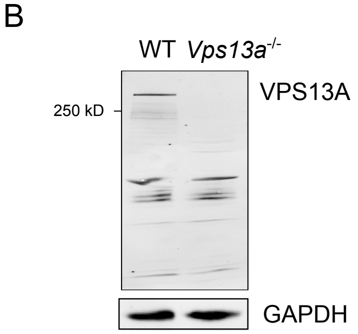

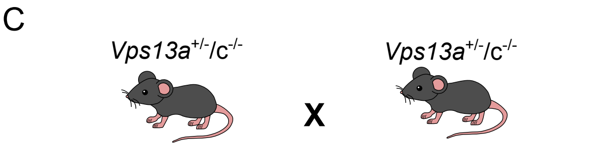

**Age**	*Vps13a*$^{+/+}$/*c*$^{-/-}$	*Vps13a*$^{+/-}$/*c*$^{-/-}$	*DKO*	**Total**
Adult (>3 week)	25 (19)	50 (38)	0 (19)	75
E12.5	12 (12)	21 (25)	16 (12)	49

bioRxiv preprint doi: https://doi.org/10.1101/2025.05.09.653147; this version posted May 13, 2025. The copyright holder for this preprint (which was not certified by peer review) is the author/funder, who has granted bioRxiv a license to display the preprint in perpetuity. It is made available under aCC-BY 4.0 International license.

Fig. S2

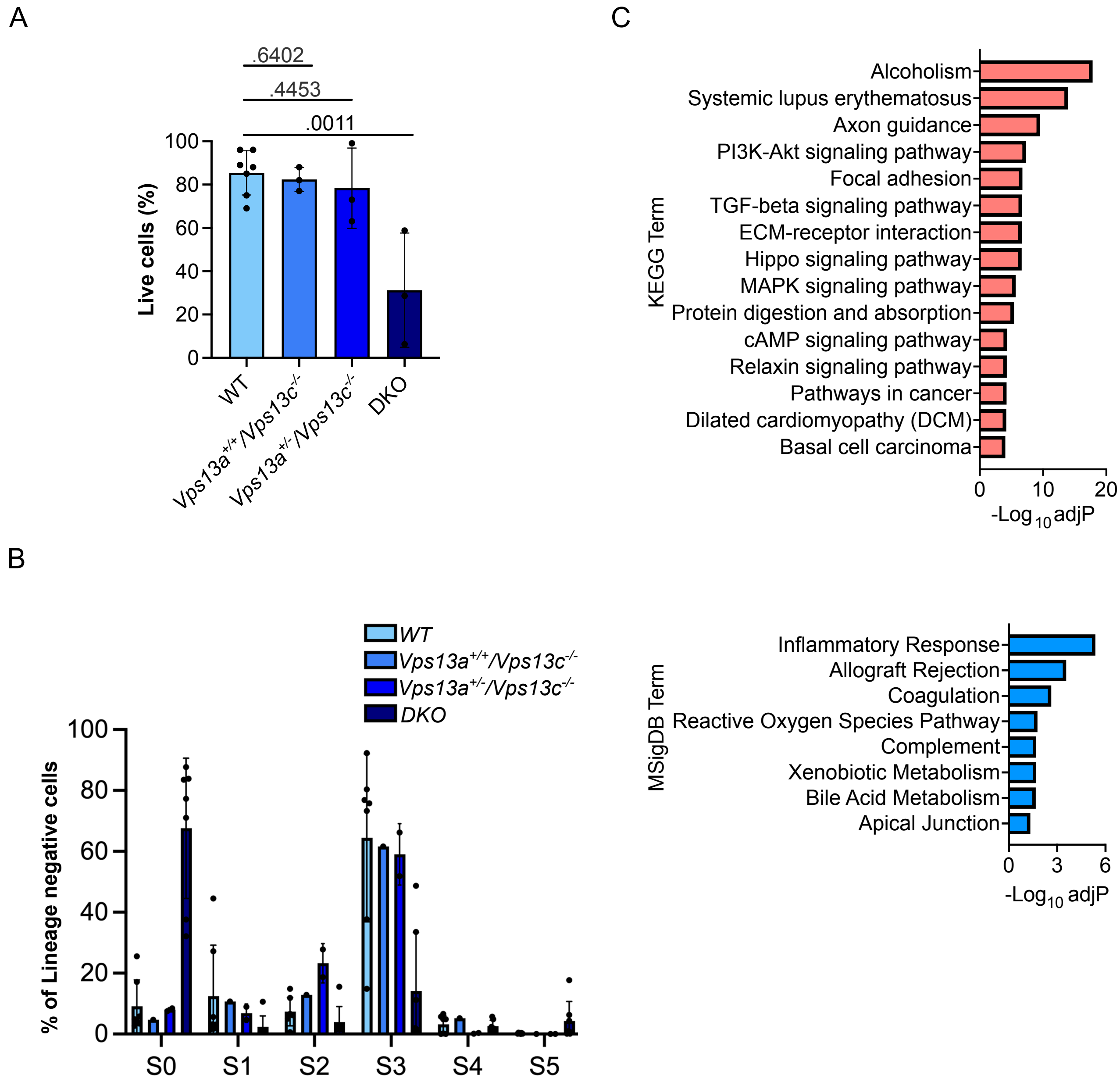

Supplement: Supplement 1 [file NIHPP2025.05.09.653147v1-supplement-1.pdf]
